# Comparison of Praat and Computerized Speech Lab for formant analysis of five Japanese vowels in maxillectomy patients

**DOI:** 10.3389/fnins.2023.1098197

**Published:** 2023-02-01

**Authors:** Islam E. Ali, Yuka Sumita, Noriyuki Wakabayashi

**Affiliations:** ^1^Department of Advanced Prosthodontics, Graduate School of Medical and Dental Sciences, Tokyo Medical and Dental University, Tokyo, Japan; ^2^Department of Prosthodontics, Faculty of Dentistry, Mansoura University, Mansoura, Egypt

**Keywords:** Praat, Computerized Speech Lab (CSL), maxillectomy, vowels, formant analysis

## Abstract

**Introduction:**

Speech impairment is a common complication after surgical resection of maxillary tumors. Maxillofacial prosthodontists play a critical role in restoring this function so that affected patients can enjoy better lives. For that purpose, several acoustic software packages have been used for speech evaluation, among which Computerized Speech Lab (CSL) and Praat are widely used in clinical and research contexts. Although CSL is a commercial product, Praat is freely available on the internet and can be used by patients and clinicians to practice several therapy goals. Therefore, this study aimed to determine if both software produced comparable results for the first two formant frequencies (F1 and F2) and their respective formant ranges obtained from the same voice samples from Japanese participants with maxillectomy defects.

**Methods:**

CSL was used as a reference to evaluate the accuracy of Praat with both the default and newly proposed adjusted settings. Thirty-seven participants were enrolled in this study for formant analysis of the five Japanese vowels (a/i/u/e/o) using CSL and Praat. Spearman’s rank correlation coefficient was used to judge the correlation between the analysis results of both programs regarding F1 and F2 and their respective formant ranges.

**Results:**

As the findings pointed out, highly positive correlations between both software were found for all acoustic features and all Praat settings.

**Discussion:**

The strong correlations between the results of both CSL and Praat suggest that both programs may have similar decision strategies for atypical speech and for both sexes. This study highlights that the default settings in Praat can be used for formant analysis in maxillectomy patients with predictable accuracy. The proposed adjusted settings in Praat can yield more accurate results for formant analysis of atypical speech in maxillectomy cases when the examiner cannot precisely locate the formant frequencies using the default settings or confirm analysis results obtained using CSL.

## 1. Introduction

Digital acoustic analysis is particularly important in evaluating typical and atypical speech in different contexts, including clinical assessment, research, and education. Maxillectomy surgery for cancer treatment often results in impaired speech intelligibility due to unfavorable communication between the oral and nasal cavities (Sumita et al., 2018; Fadhil and Mumcu, 2019). In other words, the structure of the vocal tract is changed by the surgical operation, thereby causing vowel disarticulation. The communication between the oral and nasal cavities lowers the intraoral air pressure, which is required for speech production, and leads to hypernasal speech, nasal air emission, and reduced loudness (Babu et al., 2020).

Vowels have special importance in speech acoustics and are realized as practical representations of general speech. Among phonetic sounds, vowels are the simplest sounds to analyze and describe acoustically without the need for an anechoic chamber (Sumita et al., 2002). In addition, the correlation between vowel quality and speech intelligibility has been reported in previous studies (Sumita et al., 2002, 2018; Elbashti et al., 2019). Therefore, the acoustic analysis of vowels is considered a helpful tool for evaluating speech production in maxillectomy patients as well as in other forms of atypical speech. This is because changes in the shape of the vocal tract alter its resonance properties, affecting the quality of the produced vowels and the clarity of consonant perception (Kent et al., 2002). Therefore, speech rehabilitation after prosthetic treatment is necessary to help these patients reintegrate into society. One type of acoustic analysis that can be related to the movement of the articulators in speech is measurement of formant frequency. Formants are meaningful frequency components of human articulation that reflect the vocal tract’s resonance characteristics. The first two formants, F1 and F2, are typically used for vowel disambiguation and determining vowel quality in terms of the open/close and front/back dimensions (Sumita et al., 2002, 2018).

However, the values of acoustic measurements are presented differently in different acoustic analysis software packages (AASPs) (Deliyski et al., 2005; Smits et al., 2005; Woehrling and de Mareüil, 2007; Burris et al., 2014). This is due to differences in the algorithmic architectures of each program. Computerized Speech Lab (CSL) and Praat are two widely used AASPs in clinical and research contexts, and both calculate a series of parallel acoustic measures (Burris et al., 2014). Furthermore, they can be easily compared because they have the option of averaging the formant value across the vowel over a certain length of time. CSL is the most sophisticated system for voice and speech analysis. It is a high-performance commercial product that has all the necessary hardware and software components, including an input/output recording device for a computer with unique characteristics for accurate acoustic measurements. Praat is freely available online and was developed to enable the creation of multi-platform applications with scripting languages. Additionally, patients can download the program to their computer and use it to practice various therapy goals.

Although the default settings of AASPs are easy to use by people without much experience in speech acoustics and are often preferred by clinicians for faster acoustic analysis, on some occasions, changing the default settings is necessary to improve analysis results, particularly for atypical speech. When used to analyze normal adult speech with the manufacturers’ default settings, Praat generates formant frequency measurements that are reliable and comparable to manual measurements obtained using CSL (Burris et al., 2014). However, in the case of atypical speech in maxillectomy patients, there is no guarantee that the default settings can be used with predictable accuracy. In contrast, manual measurements of vowel formants in CSL are reported to correlate strongly with the speech intelligibility scores of maxillectomy patients when the default settings are adjusted for analysis (Sumita et al., 2018; Elbashti et al., 2019). In the present study, the CSL settings were adjusted based on recommendations from a previous study (Sumita et al., 2018), and the acoustic features extracted from the five Japanese vowels in maxillectomy patients were compared with those measured using both the default and adjusted settings for Praat. The proposed adjusted settings were intended to improve the formant analysis results of atypical speech in maxillectomy patients. We aimed to determine whether CSL and Praat with the default and adjusted settings offer comparable results for formant analysis of the same voice samples in Japanese participants with maxillectomy defects.

## 2. Materials and methods

### 2.1. Participants

Participants were 37 maxillectomy patients [22 men, 15 women; average age, 52.5 (range 30–75) years] who were treated at the Clinic of Maxillofacial Prosthetics at Tokyo Medical and Dental University and satisfied the inclusion criteria. All participants were native Japanese speakers with normal hearing and no observable abnormalities of the vocal cords. Patients who had a recurrence of oral cancer, history of neurological problems, hearing problems, or abnormality related to the vocal cords were excluded. Aramany Class I maxillectomy had been performed in 17 patients (Aramany, 1978), Aramany Class II maxillectomy in 12, and Aramany Class IV maxillectomy in 8. The patients were prosthetically rehabilitated at our clinic using different types of obturator prostheses depending on their oral conditions and were using the prosthesis for 3–5 years after surgery. The testing for formants was only done if the patients were comfortable with their prosthesis after professional adjustments.

This study was approved by the Ethics Committee of the Faculty of Dentistry, Tokyo Medical and Dental University (approval # D2022-4) and all patients agreed to participate in this study.

### 2.2. Speech samples

Voice samples of the five Japanese vowels (a/i/u/e/o) were obtained from all participants in a sound-treated room using a high-quality dynamic microphone (SM48; Shure, Tokyo, Japan) placed 20 cm away from the lips with a sampling rate of 44,100 Hz, which is the finest frequency used for recording most sounds (Gupta et al., 2022). During vowel recording, participants were instructed to take a short breath and hold the vowel sound for 3–4 s. The participants made consistent and distinct vowel sounds at a conversational volume level and could see a display indicating the sound level in decibels (dB). To decrease the effect of the previously spoken vowel, the participants paused between each utterance for 1 or 2 s. Moreover, each participant was instructed to try to maintain the same vowel quality throughout the entire utterance (Sumita et al., 2002). An investigator demonstrated the procedure before beginning the recording, and participants completed practice trials until they demonstrated a complete understanding of the task. Voice samples were saved in.wav format, which is compatible with both CSL and Praat.

### 2.3. Acoustic analysis

The acoustic analysis was performed by one operator using both software. The middle 1 s (s) of each voice sample was always selected for analysis and an automatic mode of formant retrieval was applied to minimize the recording observation bias. A further confirmatory step was used to detect formants manually, and a second operator was consulted whenever the results of both modes showed marked discrepancy. The settings applied for both software were as follows:

#### 2.3.1. Acoustic analysis using CSL

The acoustic analysis settings in CSL (CSL 4400; KayPentax, Lincoln Park, NJ, USA) were adjusted based on a previous study on maxillectomy patients (Table 1; Sumita et al., 2018). For acoustic analysis, the middle 1 s of each vowel was selected because this is usually when formant tracks are most stable. The formant history (automatic) and linear predictive coding (LPC, manual) were jointly utilized to retrieve the formant data for each vowel. For each participant, the mean values of F1 and F2 across the selection were recorded from the formant history interface for each of the five vowels. An autocorrelation approach for LPC was used to calculate the formant frequencies for further confirmation of the formant history results. The LPC spectra displayed the vowel’s amplitude by frequency. F1 and F2 are the frequencies of the first two spectral peaks retrieved from the LPC spectra. The same procedures were repeated for all participants to obtain the raw data used for statistical analysis.

**TABLE 1:** The settings of Computerized Speech Lab (CSL) and Praat used for formant analyses in this study.

Software	Formant ceiling	Number of formants	Window length	LPC order (number of coefficients)	Pre-emphasis	Window type
CSL_(adjusted)	4,410 Hz for formant history	4–5 formants depending on filter order.	30 ms	12 for males 12–16 for females	0.9 for formants	Hamming
Praat_(default)	5,500 Hz	5	25 ms	10	From 50 Hz	Gaussian
Praat_(adjusted)	4,000 Hz for males 4,600 Hz for females	4	40 ms	8	From 50 Hz	Gaussian

The following criteria were always considered to decide the LPC filter order for estimating formant data of a given vowel:

a. The congruence of the displayed LPC- derived formant tracks and the corresponding dark bands in the spectrogram.
b. The continuity of the formant track across the vowel duration.
c. The agreement between the data of the automatic mode formant history) and manual mode LPC) of formant estimations.
d. The expected formant values for a given speaker based on normative data, considering the gender, age, and the vowel being evaluated (Duckworth et al., 2011; Derdemezis et al., 2016).
e. The bandwidth of the LPC spectral peak of the formant of interest; (rejected if greater than 500 HZ) (Derdemezis et al., 2016).
f. If the LPC analysis appears to miss a formant, as in the case of F1 and F2 for back vowels, the analysis was improved by increasing the filter order in small increments.

Taking the above criteria into account (Duckworth et al., 2011; Derdemezis et al., 2016), a range of 12–16 of filter order was empirically found to be the most suitable for formant estimation in female speakers with maxillectomy defects following a trial-and-error process. For male speakers, a filter order of 12 has already been documented in a previous work by Sumita et al. (2002).

#### 2.3.2. Acoustic analysis using Praat default settings

**FIGURE 1:**
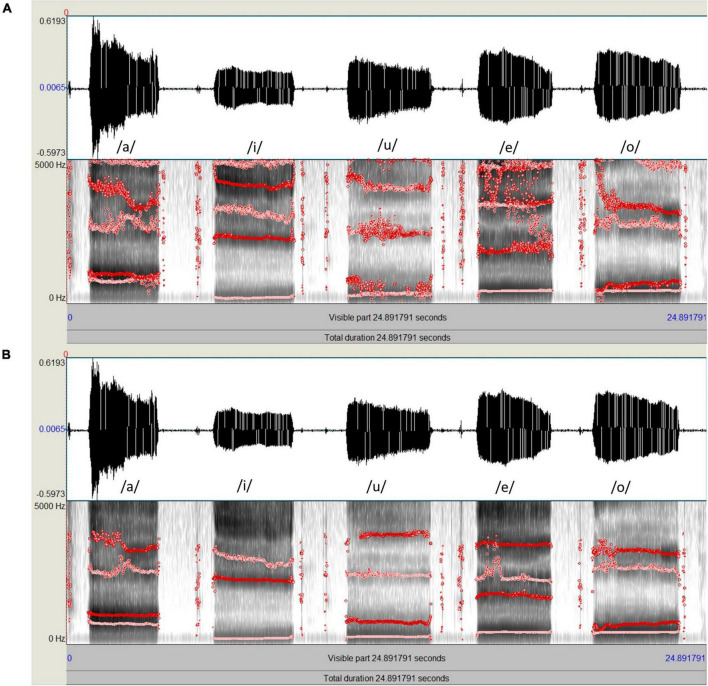
Spectrograms and formant tracks of the same speech sample of five Japanese vowels (a/i/u/e/o); (**A**, Top) Formant analysis using Praat with the default settings. (**B**, Bottom) Formant analysis using Praat with adjusted settings, which resulted in more stable formant tracks.

The waveform files of all participants that were recorded using CSL were imported into Praat (Version 6.2.09; developed by Boersma and Weenink, Institute of Phonetic Sciences, University of Amsterdam, Netherlands). Each recording was viewed separately for formant analysis. The Praat software was used without changing the default settings (Table 1). The formant ceiling was 5,500 Hz, and five formants were displayed (Figure 1A) with a window length of 0.025 s and a dynamic range of 30 dB. To calculate the formants for each participant, a 1s segment was extracted from the middle portion of the amplitude-by-time waveform, and F1 and F2 formants for each vowel were automatically calculated using the pre-set “Log 2” script from the “query” menu of the software. By this script, the mean values of the first and second formants across the selection are retrieved and rounded up to whole numbers in Hz. The results were further confirmed by manual measurement using the LPC spectrum of the selected spectral slice. The same procedures were repeated for all participants to obtain the raw data used for statistical analysis.

#### 2.3.3. Acoustic analysis using Praat adjusted settings

For acoustic analysis using Praat, the waveform file of each speech sample was viewed on a computer monitor. The formant ceiling was set to 4,000 Hz for men and 4,600 Hz for women. The number of formants displayed was set to a maximum of four using a window length of 0.04 s and a dynamic range of 30 dB. Empirical observations using the aforementioned settings produced clearly defined peaks on the spectrogram and stable formant tracks (Figure 1B). To calculate the formants for each participant, a 1s segment was extracted from the middle portion of the amplitude-by-time waveform, and the mean F1 and F2 for each vowel across the selection were automatically calculated using the pre-set “Log 2” script from the “query” menu of the software. The results were further confirmed by manual measurement using the LPC spectrum of the selected spectral slice. The same procedures were repeated for all participants to obtain the raw data used for statistical analysis.

### 2.4. F1 and F2 range calculation

The ranges of F1 and F2 are valuable markers for speech perception and speech intelligibility (Bradlow et al., 1996; Elbashti et al., 2019). To determine the F1 range for each speaker, the difference between the maximum and minimum F1 frequencies of the five vowels uttered by that speaker was calculated. Similarly, the F2 range for each speaker was determined by calculating the difference between the maximum and minimum F2 frequencies of the five vowels uttered by that speaker.

### 2.5. Statistical analysis

For both CSL and Praat, the distributions were significantly non-normal for the F1 and F2 data and their respective formant ranges (*p* < 0.05) according to the Shapiro–Wilk test (Tables 2, 3). Based on this outcome, and after a visual examination of the histograms and Q-Q plots (quantile-quantile plots) (Das and Imon, 2016), a non-parametric test was used. Spearman’s rank correlation coefficient (rs) was calculated to assess the relationship between the formant data (F1 and F2) as well as the formant ranges (F1 and F2 ranges) obtained by Praat and those obtained by CSL for all settings. A *P*-value of < 0.05 was considered to indicate statistical significance. All analyses were performed using JMP ver. 16 (JMP Statistical Discovery LLC, Cary, NC, USA).

**TABLE 2:** Summary statistics and normality test results for F1 and F2 data obtained using Computerized Speech Lab (CSL) and Praat for all settings.

Variable	Min.	Max.	Median	Interquartile range	Shapiro–Wilk	
					W	*P*-value
F1_CSL (adjusted)	177	1,013	408	212.5	0.919	*p* < 0.0001*
F1_Praat (default)	194	955	437	215	0.945	*p* < 0.0001*
F1_Praat (adjusted)	197	1,022	428	208.5	0.929	*p* < 0.0001*
F2_CSL (adjusted)	478	2,966	1,191	1038.5	0.924	*p* < 0.0001*
F2_Praat (default)	695	2,974	1,415	1,005	0.943	*p* < 0.0001*
F2_Praat (adjusted)	480	2,971	1,298	1042.5	0.934	*p* < 0.0001*

*A *P*-value less than 0.05 is considered statistically significant.

**TABLE 3:** Summary statistics and normality test results for F1 and F2 ranges obtained using Computerized Speech Lab (CSL) and Praat for all settings.

Variable	Min.	Max.	Median	Interquartile range	Shapiro-Wilk	
					W	*P*-value
F1 range_CSL (adjusted)	160	745	364	150	0.935	*P* = 0.034*
F1 range_Praat (default)	180	673	394	181.5	0.983	*p* = 0.839
F1 range_Praat (adjusted)	126	751	372	133	0.937	*p* = 0.039*
F2 range_CSL (adjusted)	455	2,440	1,445	604	0.983	*p* = 0.043*
F2 range_Praat (default)	522	2,214	1,416	414.5	0.963	*p* = 0.244
F2 range_Praat (adjusted)	312	2,236	1,462	554	0.979	*p* = 0.700

*A *p*-value less than 0.05 is considered statistically significant.

## 3. Results

**FIGURE 2:**
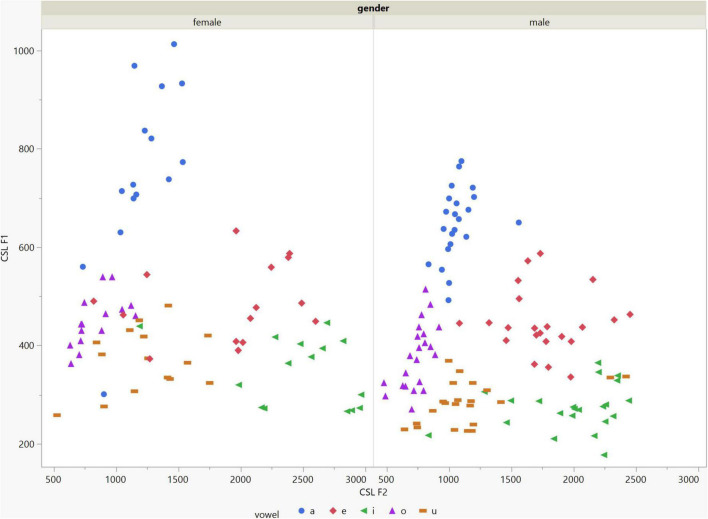
Arrangement of the F1 and F2 data of the five vowels calculated using Computerized Speech Lab (CSL) for both male and female patients. The data for females showed more scatter along the F1 and F2 axes compared with the data for males specially for the /a/, /i/, /e/ vowels.

**FIGURE 3:**
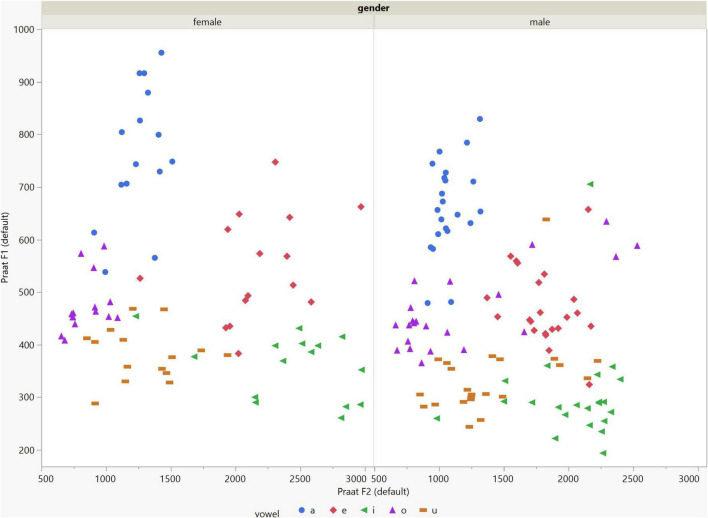
Arrangement of the F1 and F2 data of the five vowels calculated using Praat with the default settings for both males and females.

**FIGURE 4:**
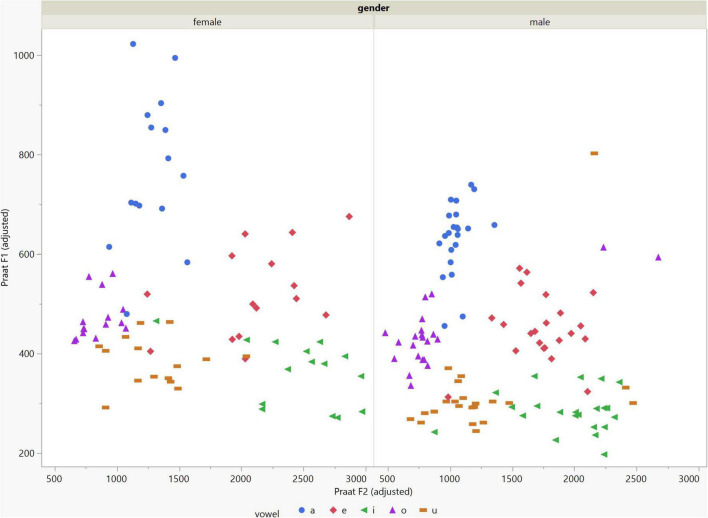
Arrangement of the F1 and F2 data of the five vowels calculated using Praat with the adjusted settings. The data for females showed more scatter along the F1 and F2 axes compared with the data for males.

**FIGURE 5:**
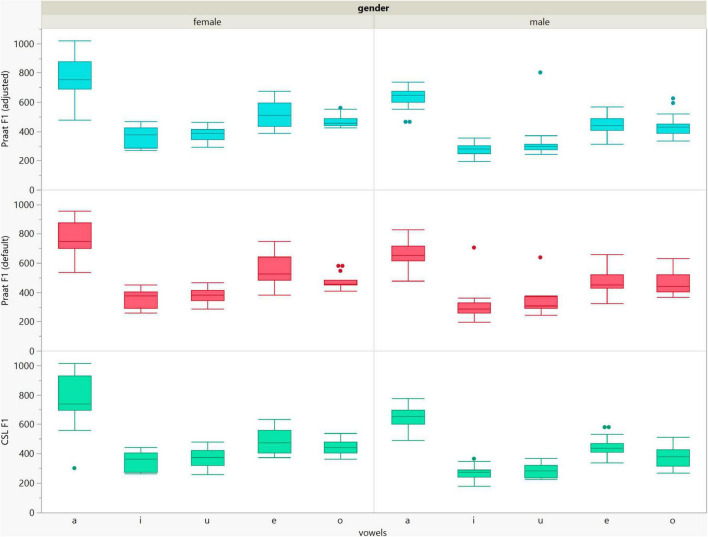
Box plots of the F1 data of the five vowels calculated using Computerized Speech Lab (CSL) and Praat with the default and adjusted settings for both men and women.

**FIGURE 6:**
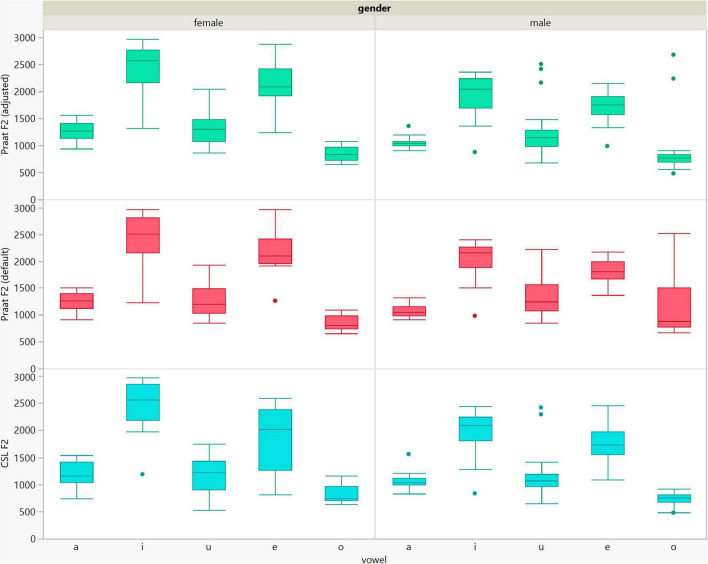
Box plots of the F2 data of the five vowels calculated using Computerized Speech Lab (CSL) and Praat with the default and adjusted settings for both men and women.

**FIGURE 7:**
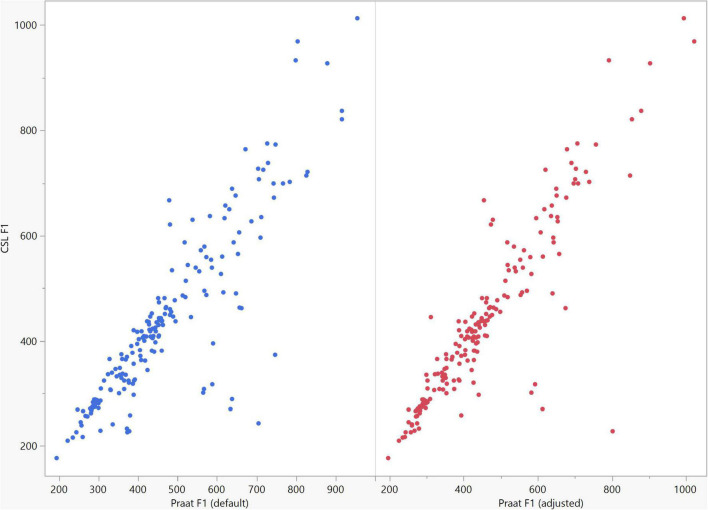
Correlation between the F1 data of the five vowels calculated using Computerized Speech Lab (CSL) and Praat. The CSL F1 data showed strong positive correlations with those calculated by Praat for both the default (rs = 0.867, *n* = 185, *p* < 0.0001*) and adjusted (rs = 0.894, *n* = 185, *p* < 0.0001*).

**FIGURE 8:**
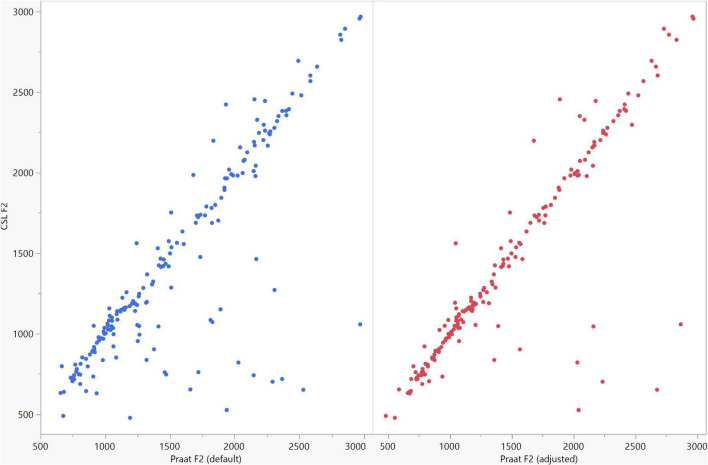
Correlation between the F2 data of the five vowels calculated using Computerized Speech Lab (CSL) and Praat. The CSL F2 data showed strong positive correlations with those calculated by Praat for both the default (rs = 0.785, *n* = 185, *p* < 0.0001*) and adjusted (rs = 0.873, *n* = 185, *p* < 0.0001*) settings.

**FIGURE 9:**
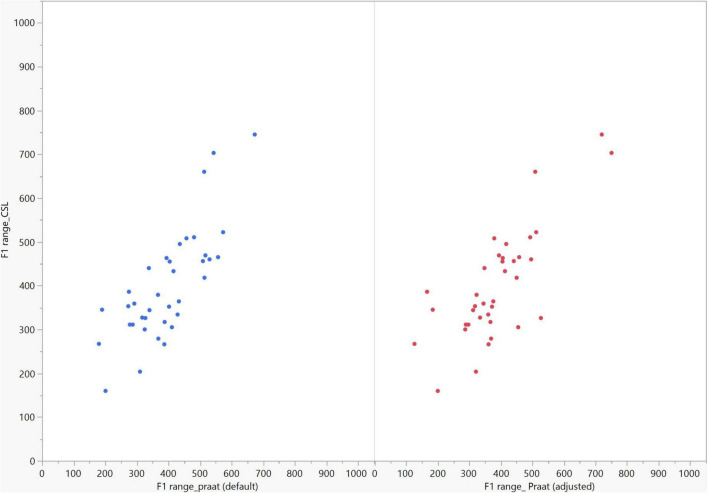
Correlation between the F1 ranges of five vowels calculated from Computerized Speech Lab (CSL) F1 data and Praat F1 data using the default and adjusted settings. The calculated F1 ranges were highly correlated between CSL and both Praat settings (default settings: rs = 0.752, *p* < 0.0001*; adjusted settings: rs = 0.708, *n* = 37, *p* < 0.0001*).

**FIGURE 10:**
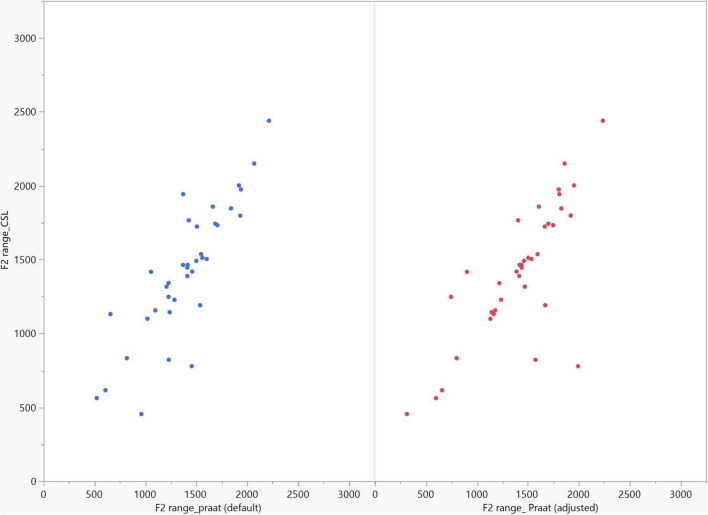
Correlation between the F2 ranges of five vowels calculated from Computerized Speech Lab (CSL) F2 data and Praat F2 data using the default and adjusted settings. The calculated F2 ranges were highly correlated between CSL and both Praat settings (default settings: rs = 0.850, n = 37, *p* < 0.0001*; adjusted settings: rs = 0.732, *n* = 37, *p* < 0.0001*).

In general, the F1 and F2 frequencies calculated using Praat with the adjusted settings had a higher correlation with those obtained from CSL (Table 4) by controlling the displayed formants and providing smoother formant tracks. The arrangement of the formant data (F1 and F2) of the five vowels calculated by CSL and Praat for both men and women are shown in Figures 2–4. Generally, the data for women showed more scatter along the F1 and F2 axes compared with the data for men regardless of the program used. Among the five vowels, the F1 and F2 of three vowels (a/i/e) were the sources of greatest scatter in the data for women suggesting that these vowels are the least controlled by female patients after maxillectomy (Figures 5, 6). It might also imply that the settings of the software should be adjusted based not only on gender but also on the vowel being analyzed. Meanwhile, more outliers were evident in men’s data compared to females’ (Figures 5, 6). The Spearman correlation is less sensitive to strong outliers because it limits the outlier to the value of its rank. In addition, a robust estimation was used to control outliers by down-weighting extreme values. The CSL F1 data showed strong positive correlations with those calculated by Praat in both the default (rs = 0.867, *n* = 185, *p* < 0.0001*) and adjusted (rs = 0.894, *n* = 185, *p* < 0.0001*) settings (Figure 7). Similarly, the CSL F2 data showed strong positive correlations with those calculated by Praat for both the default (rs = 0.785, *n* = 185, *p* < 0.0001) and adjusted settings (rs = 0.873, *n* = 185, *p* < 0.0001*) settings (Figure 8). The calculated ranges for F1 were highly correlated between CSL and both Praat settings (default settings: rs = 0.752, *n* = 37, *p* < 0.0001*; adjusted settings: rs = 0.708, *n* = 37, *p* < 0.0001*; Table 5 and Figure 9). The calculated ranges for F2 were also highly correlated between CSL and both Praat settings (default settings: rs = 0.850, *n* = 37, *p* < 0.0001*; adjusted settings: rs = 0.732, *n* = 37, *p* < 0.0001*; Table 5 and Figure 10).

**TABLE 4:** Spearman’s rank correlations of F1 and F2 data of the five Japanese vowels calculated by Computerized Speech Lab (CSL) to those calculated using the default and adjusted settings of Praat.

Variable	By variable	Spearman’s correlation coefficient (rs)	*P*-value	N
Praat F1 (default)	CSL F1	0.8674	<0.0001*	185
Praat F1 (adjusted)	CSL F1	0.8944	<0.0001*	185
Praat F2 (default)	CSL F2	0.7846	<0.0001*	185
Praat F2 (adjusted)	CSL F2	0.8727	<0.0001*	185

*A *P*-value of < 0.05 indicates statistical significance.

**TABLE 5:** Spearman’s rank correlation of F1 and F2 ranges of the five Japanese vowels calculated by Computerized Speech Lab (CSL) to those calculated using the default and adjusted settings of Praat.

Variable	By variable	Spearman’s correlation coefficient (rs)	*P*-value	N
F1 range_Praat (default)	F1 range_CSL	0.752	<0.0001*	37
F1 range_Praat (adjusted)	F1 range_CSL	0.708	<0.0001*	37
F2 range_Praat (default)	F2 range_CSL	0.850	<0.0001*	37
F2 range_Praat (adjusted)	F2 range_CSL	0.732	<0.0001*	37

*A *P*-value of < 0.05 indicates statistical significance.

## 4. Discussion

In our study, the results of acoustic analysis of the five Japanese vowels using CSL and Praat were highly correlated for the first and second formants (F1 and F2) and their respective ranges. Strong correlations between the results of both CSL and Praat suggest that both programs may have similar decision strategies for pathological voices and for both sexes.

The benefits of Praat are its ability to annotate and label speech files, which simplifies analysis, and its voice report function. Meanwhile, CSL supports the usage of other modules such as the Multi-Dimensional Voice Profile (MDVP) module, which enables a detailed analysis of the speaker’s voice. All of these features have practical applications depending on the research and clinical situation (Burris et al., 2014). The choice of data-gathering tools and acoustic-analysis programs used in a voice clinic may be influenced by a variety of different circumstances, including patient turnover, the staff skill level, and budgetary restrictions. In addition, the decision about which AASP to use should consider the user’s needs, their knowledge of and familiarity with acoustic analysis and acoustic science, and the specific analysis objectives.

Burris et al. (2014) reported that the default settings in CSL are not optimal for analyzing formant frequencies in women or children. Likewise, the same study showed less satisfactory results for women compared with men when formant analysis was performed using Praat with the default settings. Therefore, it is important that users be cautious and consider adjusting the settings to achieve optimal analysis results. When investigating pathological speech, it becomes more crucial to customize these settings for both men and women in order to accommodate the abnormal speech acoustics in these patients. In the present study, the CSL settings were adjusted as recommended by a previous study on patients with similar classes of maxillectomy defects (Sumita et al., 2018). Because only male patients were included in that study, a minor modification was introduced here by increasing the filter order for female patients when LPC or formant history methods were used in CSL to estimate formant frequencies. Women generally have higher-pitched voices than men. It means that fundamental frequency is higher than men, which is related to the membranous length of the vocal folds. The higher fundamental frequency of woman’s voices causes difficulties in acoustic analysis. As fundamental frequency increases, there is a corresponding increase in the interval between harmonics of the laryngeal source spectrum. At some harmonic spacing, it becomes difficult to discern the location of formants in the spectrum. By increasing the filter order, usually in steps of 2, more spectral details are resolved, at least until the additional detail begins to obscure the formant pattern (Kent and Vorperian, 2018).

Formants are estimated in Praat by first resampling to twice the ceiling of the formant search range and then applying a pre-emphasis filter, windowing the speech in the time domain using a Gaussian window, and estimating the LPC coefficients using the algorithm by Burg (Sandoval et al., 2019). The proposed adjusted settings for Praat using a longer window and a lowered formant ceiling provide more stable formant tracks and definite formant peaks for better estimation of formant frequencies in maxillectomy patients. Praat does not have an option to directly manipulate the number of coefficients. However, users can indirectly adjust the number of coefficients by changing the default number of formants as we did in this study. By decreasing the number of formants displayed from 5 (default) to 4 (adjusted), the number of coefficients is changed from 10 to 8, which is preferable given that the first four formants are of particular interest in speech research (Derdemezis et al., 2016).

In general, men usually exhibit very noticeable compacting and lowering of formant values compared with women (Coleman, 1971), which might explain the greater dispersion of data for women (Sandoval et al., 2019). Additionally, the altered oral anatomy resulting from surgery tends to decrease the formant values of both sexes compared with non-defect patients due to impaired resonance quality and increased size of the vocal tract (Elbashti et al., 2019; Fadhil and Mumcu, 2019). Therefore, by decreasing the number of displayed formants and customizing the value of the formant ceiling to accommodate sex differences and altered oral anatomy, there is less chance of mistakenly including a spurious peak as a formant peak, which improves the accuracy of obtained results. This accounts for the higher correlation between the analysis results obtained using Praat with the adjusted settings and those using CSL. The acoustic analysis results using Praat with the default settings were likewise reasonable; therefore, the default settings can still be applied to a simplified acoustic analysis if the sensitivity to changes in the formant pattern is not critical. The convenience of automatic formant measurements by AASPs needs to be weighed against the tolerance for error, which depends on the intended application and the purpose of the measurement (Derdemezis et al., 2016).

The calculation of formant ranges only considers two values, the maximum and minimum formant values among the five vowels for each patient. Therefore, it does not include information from all five vowels which might explain why correlation pattern was slightly inverted for formant ranges compared to formant data using default and adjusted settings. Another finding is that some of the F1 and F2 data did not follow the correlation line of fit between both software for both settings of Praat, therefore, a future study may be needed to clarify such finding and customize the settings for analysis based on the direction of current study.

The limitations of this study are as follows: only two acoustic features (F1 and F2) were assessed, only maxillectomy patients were investigated, the effects of patient age were not evaluated, and only two AASPs were compared. As a result, additional formants, the depth of valleys and the shift in slopes near formant peaks, patient groups other than maxillectomy cases, the effects of age, and other AASPs should be investigated in future studies. Furthermore, for the sake of comparison and to minimize the effect of the recording method, CSL was used to record all speech samples in this study because CSL includes a high-quality microphone, cable, and soundboard. Whether recording with Praat influences the obtained results needs to be confirmed by another investigation.

## 5. Conclusion

The results of digital acoustic analysis of the five Japanese vowels in maxillectomy patients were highly correlated between CSL and Praat. Therefore, it could be concluded that both programs have similar decision strategies for formant analysis of pathological voices of maxillectomy cases. Using the default settings of Praat, ageneral practitioner can benefit from this free software to monitor the effects of prosthetic treatment on speech rehabilitation without the need for special tools or a deep understanding of speech acoustics. The proposed adjusted settings for Praat can yield more accurate formant data in maxillectomy patients when the examiner cannot precisely locate the formant frequencies using the default settings and it can also be used to confirm analysis results obtained using CSL. Gender differences in speech characteristics should be always considered when adjusting the settings of both software for optimum acoustic analysis results.

## Data availability statement

The original contributions presented in this study are included in the article/supplementary material, further inquiries can be directed to the corresponding author.

## Ethics statement

The studies involving human participants were reviewed and approved by the Ethics Committee of the Faculty of Dentistry, Tokyo Medical and Dental University (approval #D2022-4). The Ethics Committee waived the requirement of written informed consent for participation.

## Author contributions

IA wrote the article and performed the data collection and the statistical analysis. YS contributed to the conception and design of the study. YS and NW drafted and revised the manuscript. All authors contributed to the article and approved the submitted version.
